# Inference of natural selection from ancient DNA

**DOI:** 10.1002/evl3.165

**Published:** 2020-03-18

**Authors:** Marianne Dehasque, María C. Ávila‐Arcos, David Díez‐del‐Molino, Matteo Fumagalli, Katerina Guschanski, Eline D. Lorenzen, Anna‐Sapfo Malaspinas, Tomas Marques‐Bonet, Michael D. Martin, Gemma G. R. Murray, Alexander S. T. Papadopulos, Nina Overgaard Therkildsen, Daniel Wegmann, Love Dalén, Andrew D. Foote

**Affiliations:** ^1^ Centre for Palaeogenetics 10691 Stockholm Sweden; ^2^ Department of Bioinformatics and Genetics Swedish Museum of Natural History 10405 Stockholm Sweden; ^3^ Department of Zoology Stockholm University 10691 Stockholm Sweden; ^4^ International Laboratory for Human Genome Research (LIIGH) UNAM Juriquilla Queretaro 76230 Mexico; ^5^ Department of Life Sciences, Silwood Park Campus Imperial College London Ascot SL5 7PY United Kingdom; ^6^ Animal Ecology, Department of Ecology and Genetics, Science for Life Laboratory Uppsala University 75236 Uppsala Sweden; ^7^ Globe Institute University of Copenhagen DK‐1350 Copenhagen Denmark; ^8^ Department of Computational Biology University of Lausanne 1015 Lausanne Switzerland; ^9^ SIB Swiss Institute of Bioinformatics 1015 Lausanne Switzerland; ^10^ Institut de Biologia Evolutiva (CSIC‐Universitat Pompeu Fabra), Parc de Recerca Biomèdica de Barcelona Barcelona Spain; ^11^ National Centre for Genomic Analysis—Centre for Genomic Regulation Barcelona Institute of Science and Technology 08028 Barcelona Spain; ^12^ Institucio Catalana de Recerca i Estudis Avançats 08010 Barcelona Spain; ^13^ Institut Català de Paleontologia Miquel Crusafont Universitat Autònoma de Barcelona Cerdanyola del Vallès Spain; ^14^ Department of Natural History, NTNU University Museum Norwegian University of Science and Technology (NTNU) Trondheim Norway; ^15^ Department of Veterinary Medicine University of Cambridge Cambridge CB2 1TN United Kingdom; ^16^ Molecular Ecology and Fisheries Genetics Laboratory, School of Biological Sciences Bangor University Bangor LL57 2UW United Kingdom; ^17^ Department of Natural Resources Cornell University Ithaca New York 14850; ^18^ Department of Biology Université de Fribourg 1700 Fribourg Switzerland; ^19^ Swiss Institute of Bioinformatics Fribourg Switzerland

**Keywords:** Adaptation, ancient DNA, natural selection, paleogenomics, time series

## Abstract

Evolutionary processes, including selection, can be indirectly inferred based on patterns of genomic variation among contemporary populations or species. However, this often requires unrealistic assumptions of ancestral demography and selective regimes. Sequencing ancient DNA from temporally spaced samples can inform about past selection processes, as time series data allow direct quantification of population parameters collected before, during, and after genetic changes driven by selection. In this Comment and Opinion, we advocate for the inclusion of temporal sampling and the generation of paleogenomic datasets in evolutionary biology, and highlight some of the recent advances that have yet to be broadly applied by evolutionary biologists. In doing so, we consider the expected signatures of balancing, purifying, and positive selection in time series data, and detail how this can advance our understanding of the chronology and tempo of genomic change driven by selection. However, we also recognize the limitations of such data, which can suffer from postmortem damage, fragmentation, low coverage, and typically low sample size. We therefore highlight the many assumptions and considerations associated with analyzing paleogenomic data and the assumptions associated with analytical methods.

**Impact Summary**

The search for signatures of natural selection on the genome is still most commonly based on screening modern genomes for regions of reduced diversity or increased differentiation between populations. This framework is essentially a snapshot in time of a process that may have played out over many millennia, during which changes in population size, ecology and gene flow between populations may have played a role in determining genetic variation. Here, we outline how utilising ancient DNA (aDNA) techniques to sequence time series of genomes spanning changes in natural selection can provide a more nuanced understanding of how natural selection has impacted genomic variation in present‐day populations. In particular, we argue that the advent of paleo‐population genomics, in which datasets of multiple individuals spanning millennia have been sequenced, offers unprecedented opportunity to estimate changes in allele frequencies through time. We outline considerations and the types of data that would be needed for the inference of positive selection on traits associated with single and many genes (polygenic), genome‐wide negative (background) selection, and balancing selection. However, we recognise that there are currently few datasets existing that are suitable for these types of investigation. There is thus a bias towards study species that have undergone strong selection over relatively recent timescales that are within the scope of aDNA, such as has occurred in domesticated species. We also detail a number of caveats associated with working with aDNA data, which is by its nature comprised of short, degraded DNA fragments, typically with a high degree of missing data and DNA damage patterns. Lastly, we highlight how the predicted move towards increasingly big datasets in aDNA studies will require the adoption of new analytical techniques and efficient data storage. Emerging developments, including the recording of genealogical variation across hundreds or thousands of individuals as tree sequences, and the increased automation of analyses through machine learning, which offer exciting new opportunities for the inference of selection from aDNA.

## Introduction

**Figure 1:**
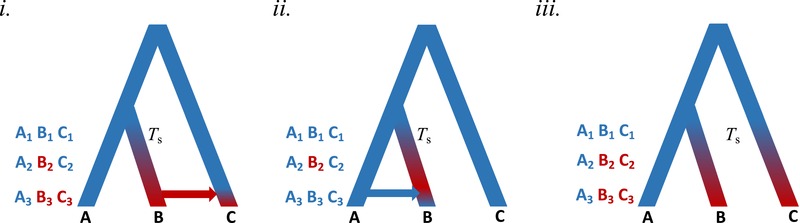
Complex demographic scenarios in which selective sweeps, due to a novel selection pressure acting upon at least one population from time *T*_s_ onward, can be masked or misinterpreted. In each scenario, sampling before (1), during (2), and after (3) *T*_s_ provides a time series of allele frequencies in populations A, B, and C, providing more power to infer the true evolutionary history. Allele frequencies are indicated by coloring of branches. *i*. Positive selection for a derived (red) allele in population B at time *T*_s_ drives it to high frequency, differentiating population B from populations A and C, but this differentiation at this locus is later masked by introgression from population B into population C. *ii*. The same evolutionary history as in *i*., except this time recent introgression of the ancestral allele (blue) from population A into population B masks the ancestral selection on the derived allele. *iii*. Parallel selection acts upon the derived allele at time *T*_s_ in both populations B and C. Three population selection tests such as the Population Branch Statistic can misinterpret this pattern of differentiation of A from both B and C as that of selection on the ancestral (blue) allele in population A (see Mathieson 2019 for an example of this type of scenario and selection on loci within the *FADS* gene in humans).

Most population genetic studies use comparisons at a single point in time or over timescales of only a few generations, and infer ancestral states using coalescent‐based methods. This snapshot of evolution may only be partially informative, as diverging populations may have experienced changes in allele frequencies due to gene flow and population size changes, which can be difficult to disentangle from signatures of natural selection (Fig. 1). Given the temporal nature of evolution, ancient DNA (aDNA) techniques are obvious and promising tools with which to track the chronology and tempo of genomic change, and thereby provide unique opportunities for detecting distinct footprints of selection. The advent and increasing efficiency of high‐throughput sequencing, combined with recent advances in aDNA extraction, library build, and data processing (Pinhasi et al. 2015; Gansauge et al. 2017; Link et al. 2017; Carøe et al. 2018; Renaud et al. 2019; Dabney and Meyer 2019; Martiniano et al. 2019; Wales and Kistler 2019), now allow the generation of paleopopulation genomic datasets, thus offering unprecedented opportunities to better understand the chronology and tempo of evolution at the genomic level.

In this review, we advocate for increased utilization of paleogenomics within the field of evolutionary biology, allowing natural selection to be investigated along the evolutionary continuum, at multiple time points throughout the process. We aim to give a nuanced discussion on the present role and future potential for aDNA data to contribute toward our understanding of selection in a broad range of organisms. We first describe how sampling across a time series can increase our understanding of the selective processes underlying patterns of genomic variation in contemporary data. We highlight the advances that have allowed the field of paleogenomics to progress over the past decade and significant challenges that remain associated with working with aDNA data. We then outline the potential and the limitations of studying different types of selection by incorporating aDNA time series data. Throughout, we try to raise awareness of the shortcomings of such data by exposing its caveats. For example, we discuss the merits of using few ancient samples for elucidating genome‐wide processes such as background selection, while acknowledging that the inference of selection will remain limited to a few key study species until sufficient sample sizes accrue. We finish with a look forward to future innovations and a summary of the state of the field.

## Temporal Sampling

The use of temporally spaced genetic data is a promising way to circumvent some of the problems inherent to methods of selection inference. The utility of analyzing time series is illustrated by “evolve and resequence” experiments combining experimental evolution under controlled laboratory or field mesocosm conditions with next‐generation sequencing. Evolve and resequence experiments have elucidated the genetic changes underlying evolution in real time over multiple generations (Long et al. 2015; Schlötterer et al. 2015; Rajpurohit et al. 2018) but are limited to species with short generation times (e.g., Turner. & Miller 2012; Bosshard et al. 2017; Good et al. 2017) and for asexually reproducing populations (Bennett et al. 1990; Baym et al. 2016; Good et al. 2017). However, due to a lack of recombination, selection dynamics in these populations cannot easily be generalized to sexually reproducing populations, which will be the main focus of this review. Furthermore, the controlled conditions of a laboratory experiment or even field mesocosm cannot capture the full complexity of evolutionary processes in the wild. Experimental populations in evolve and resequence studies can suffer from an excess of rare alleles (if sampled from large wild populations), extended linkage disequilibrium due to limited experimental population size and masking selective sweeps, and pseudoreplication (Baldwin‐Brown et al. 2014; Kelly and Hughes 2019). Studies of some natural populations have tracked the action of selection over several generations (Hendry et al. 2000; Grant and Grant 2002; Marques et al. 2018), but these remain inherently rare and limited to instances of unusually rapid evolution.

An alternative and commonly used approach to understand the temporal context of evolution in natural populations is to sample along the so‐called “speciation continuum” by comparing sister taxa at different stages of divergence from each other (Feder et al. 2012; Seehausen et al. 2014; Shaw and Mullen 2014). For instance, this approach has been applied to investigations of the accrual and erosion of genomic differentiation due to linked selection (e.g., Burri et al. 2015) and admixture (e.g., Martin et al. 2013), respectively. However, samples from natural populations along the speciation continuum are not equivalent to sampling the same population through time. Ancestral demography, differences in the presence and strength of selection pressures, and the starting substrate of standing genetic variation may be important factors to explain the variation in genomic summary statistics among populations (e.g., Fang et al. 2019; Miller et al. 2019) that are overlooked when comparing across the speciation continuum.

Sampling genomes from multiple time points in the past using aDNA techniques offers the possibility to study the chronology and tempo of natural selection across evolutionary timescales. Using genomes from the past concurrent with ecological data relevant to selection pressures, selection and its timing and strength can be inferred by directly estimating allele frequencies at each time point. It is, to some extent, analogous to “experimental evolution in the lab” and this can allow the accurate joint inference of demography and the disentangling of selection from drift in nonequilibrium populations based on differences in the rate of change in allele frequencies between selected and neutral loci (Bank et al. 2014).

## The Scope and Limitations of aDNA

aDNA has previously been defined as DNA recovered postmortem from nonideal biological material (Navascués et al. 2010). We adopt this definition, which can be applied to datasets of museum specimens spanning past decades, through to archaeological remains dated back across millennia. This material is nonideal relative to modern samples in several respects. aDNA is subject to postmortem damage, fragmentation, and decay through processes such as hydrolysis, purination, and deamination (Lindahl 1993; Allentoft et al. 2012). Although postmortem damage complicates downstream inference by introducing alleles not reflective of a sample's diversity, fragmentation imposes a theoretical limit on the age from which mappable DNA fragments can be recovered (e.g., Dabney et al. 2013; Orlando et al. 2013; Meyer et al. 2016). Despite recent advances in sequencing ultrashort DNA fragments from specimens hundreds of thousands of years old, the majority of ancient genomes sequenced to date are in the range of thousands to tens of thousands of years old (Brunson & Reich 2019; Skoglund and Mathieson 2018; Fages et al. 2019). aDNA is typically subject to contamination from external sources, reducing the ratio of endogenous to exogenous content. Of particular concern is the contamination from modern conspecific samples, which map to the reference sample alongside endogenous DNA and thus alter patterns of allele frequencies and genetic diversity. The amount of endogenous DNA surviving in museum and archaeological specimens varies among samples due to factors that include climate, substrate, and exposure to UV radiation, specimen treatment in the museums, in addition to material type. For example, dense material such as the petrous bone has been found to contain a high percentage of endogenous DNA (Pinhasi et al. 2015). Skins and pelts also have high endogenous content, but the DNA is frequently highly fragmented likely as the result of harsh chemical treatment for specimen preservation in museums (e.g., tanning; van der Valk et al. 2017). However, it is often the case that aDNA extracted from museum or archaeological specimens provides low and fragmented coverage of the genome, thereby typically limiting inference based on heterozygosity or specific loci of interest.

Populations can adapt to new selection pressures either from de novo mutations or from standing genetic variation (Barrett and Schluter 2008). Although both de novo mutations and standing variants can rise in frequency in response to selective pressures in the time window afforded by aDNA data, in the former case, selection can only act on a beneficial variant once it exists within the population. Standing genetic variation, on the other hand, is generally expected to allow a more rapid response to changes in selective pressures (Barrett and Schluter 2008). For example, recent time series studies show that adaptation from standing genetic variation can happen within only a few generations after the origin of a new selective pressure (Epstein et al. 2016; Franks et al. 2016; Marques et al. 2018). Adaptation from standing genetic variation is thus limited by the presence of genetic variation to respond to new changes, which can be dependent upon past exposure of an ancestral population to similar selective pressures (Schluter and Conte 2009; Marques et al. 2019) and the overall effective population size. Similarly, much of the genetic substrate contributing toward deleterious recessive mutation load and thereby subject to negative selection is also thought to be maintained as standing variation in heterozygous genotypes (Peischl and Excoffier 2015).

Although our ability to push the limits of aDNA retrieval and sequencing now extends to samples dating hundreds of thousands of years in age, due to the difficulties of working with aDNA detailed above, compiling population datasets of time series data from which allele frequencies can be estimated is limited to more recent timescales (up to tens of thousands of years). Thus, both the temporal scales over which aDNA datasets are likely to span, and the frequency with which both positive and negative selection acts upon standing genetic variation relative to de novo mutations, make standing variation the more tractable genetic substrate to study the effects of selection using aDNA data.

There are few existing paleogenomic datasets consisting of multiple individuals that span temporal changes in selection pressures. The most compelling findings of selection are from rich datasets associated with recent and artificially strong selective regimes, such as domestication processes, incorporating pre‐ and early domestication samples (see Irving‐Pease et al. 2018 for a review). Such studies have been conducted on domestic species including horses (Librado et al. 2017), maize (Ramos‐Madrigal et al. 2016), and dogs (Ollivier et al. 2016). The application of the methods outlined in this review to natural populations remains a rarity.

## Detecting Positive Selection on a Monogenic or Oligogenic Trait

Positive selection acting upon a single (monogenic) or few genes (oligogenic) and sites linked to the targets of selection causes the selected allele(s) at linked sites to rise to high frequency within the population. This reduces genetic diversity within the region of the genome linked to the gene(s) targeted by selection and increases differentiation and lineage sorting of these genomic region in comparison with other populations. Studies sampling contemporary populations can therefore detect positive selection on monogenic or oligogenic traits by investigating patterns of coalescence (e.g., Hermisson and Pennings 2017), measures of population differentiation such as *F*_ST_ (Lewontin and Krakauer 1973; Beaumont 2005), patterns in the site frequency spectrum (Tajima 1989; Fay and Wu 2000), or the extent of linkage disequilibrium (Kim and Nielsen 2004). However, contemporary data represent only a single point in time. A major challenge is to disentangle the various effects upon the genome of ancient population structure, positive and background selection, and nonequilibrium demography. Selection and demographic bottlenecks can leave similar patterns of genomic variation, including reduced genetic diversity in affected genomic regions, which increases lineage sorting and differentiation between populations with different demographic and evolutionary histories (Charlesworth et al. 1993; Zeng et al. 2006; Crisci et al. 2012; Li et al. 2012; Comeron 2014).

Changes in ecological conditions, geographic distribution, rates of gene flow, and population size can all influence the strength and consistency of selection, and may thus heavily confound selection estimators. High *F*_ST_ values, for instance, can be indicative of an ancestral selective sweep, but may also be caused by demographic processes (Nielsen 2005; Excoffier et al. 2009) or background selection (Cruickshank and Hahn 2014; Burri 2017). A recent study estimated that more than 95% of the human genome is affected by background selection or biased gene conversion, and thus is evolving in a nonneutral manner (Pouyet et al. 2018). Furthermore, parallel adaptation for a derived haplotype at a specific locus in two populations can be misinterpreted as selection on the ancestral haplotype in a third population in three‐population comparisons, such as implemented in the population branch statistic (see Mathieson 2019; Fig. 1). Current attempts to account for these confounding factors using only contemporary samples are either limited to simple models or rely on strong assumptions about the strength of selection and distribution of beneficial variants (Li et al. 2012). Finally, there are limitations to how far back in the past applying coalescent approaches to only contemporary samples can reach, due to lineages coalescing in ancestral bottlenecks and selection events. Inferences about historic periods of selection may therefore be restricted to relatively recent time scales and will not span all historical changes in selective pressure, for example, shifts in the selective regime associated with strong demographic founder effects during the colonization of new habitats.

Allele frequencies inferred from aDNA from a time series of samples with known ecological context can be used to infer selection, while controlling for many of these confounding factors (Bank et al. 2014; Malaspinas 2016). The foundations for inferring the underlying mode of evolution (i.e., under neutrality or selection) from time series allele frequency data are based upon the Wright‐Fisher model. The model was named after Sewall Wright and Ronald Fisher, who famously debated the extent to which drift or selection was the driving evolutionary forces underlying fluctuations in color polymorphism frequency in a time series dataset collected from a scarlet tiger moth (*Panaxia dominula*) population (Fisher and Ford 1947; Wright 1948). The Wright‐Fisher model is a simple approximation of genetic drift in a population of constant size (*N* diploid individuals) with nonoverlapping generations, in which alleles are randomly sampled from the previous generation.

**Figure 2:**
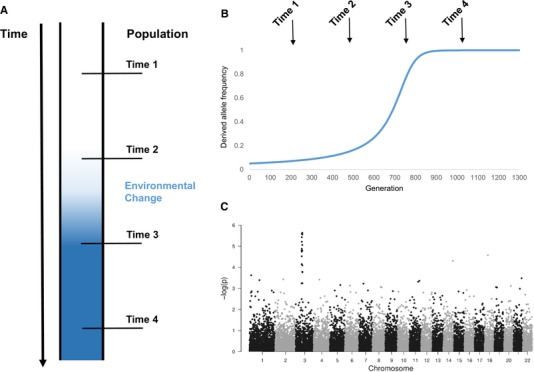
Illustration of how to track genetic adaptation of a population to environmental change through time. (A) One way to catch genetic adaptation in the act is by sampling genetic data of a population before and after the introduction of a new selective pressure (time 2 and 3, respectively). (B) Conceptual illustration of how the frequency of an allele can change in response to a new selective pressure. (C) Significant changes in allele frequencies between different populations (i.e., at time 2 and 3) can be measured with a genome‐wide scan for selection (the figure was created using the gwasResults dataframe included in qqman package in R; Turner 2014).

There are several available methods for inferring selection as a cause of directional allele frequency shift with a trajectory that is inconsistent with neutral evolution under a Wright‐Fisher model (Bollback et al. 2008; Malaspinas et al. 2012; Feder et al. 2014; Foll et al. 2015; Gompert 2016; Ferrer‐Admetlla et al. 2016; Schraiber et al. 2016). Malaspinas (2016) provided a dedicated review of how these methods work and what differentiates them from each other. These methods can then characterize selective sweeps in terms of timing, duration, and the strength of selection measured as selection coefficients (see Fig. 2; Bank et al. 2014; Malaspinas 2016). The different statistical methods using time series data to infer selection mainly differ in the statistical approach used to estimate allele frequency probabilities. As a result, different methods are suitable for different study systems, depending on the population size, the magnitude of the selection coefficient, and the parameter set to be inferred (see Malaspinas 2016). Available methods vary in their underlying assumptions and the variables that they are able to estimate. For example, some estimators can jointly infer allele age (Malaspinas et al. 2012; Schraiber et al. 2016) or population size and selection coefficients (Foll et al. 2015; Ferrer‐Admetlla et al. 2016; Schraiber et al. 2016) and account for variation in the strength of selection through time (Shim et al. 2016). However, it is important to note that most of these methods are unable to distinguish between direct and linked selection (Bank et al. 2014): they measure the by‐product of a sweep, which is the directional changes in allele frequencies at both the target and linked sites, but do not necessarily identify the target of selection if that is unknown a priori.

**Figure 3:**
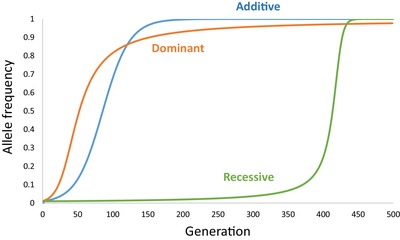
Theoretical allele trajectories under directional selection for a dominant, additive, and recessive advantageous allele. The fitness (*W*) of the different genotypes (*W*_11_, *W*_12_, *W*_22_) is defined as *W*_11_ = *W*_12_ > *W*_22_ for a dominant, *W*_11_ > *W*_12_ > *W*_22_ for an additive, and *W*_11_ > *W*_12_ = *W*_22_ for a recessive advantageous allele (allele trajectories were simulated using custom R code; R Core Team 2019).

Despite the availability of several methods for inferring selection from time series datasets, their application to aDNA datasets from natural populations remains limited (see Table S1 for an overview). Examples of applications to aDNA datasets have typically been on human‐induced selection during domestication (Ludwig et al. 2009; da Fonseca et al. 2015) or selection in humans due to dietary changes associated with domestication (Sverrisdóttir et al. 2014; Mathieson et al. 2015; Buckley et al. 2017; Ye et al. 2017; Mathieson and Mathieson 2018; Mathieson 2019). The paucity of application of such methods to aDNA datasets may reflect the scarcity of available time series of allele frequency data from aDNA, but also restrictive assumptions of the underlying Wright‐Fisher model, in particular the effect of migration on allele frequencies. This last point can to some extent be accounted for by considering a spatially structured framework in which selection coefficients and migration rates between demes can be allowed to vary (Mathieson and McVean 2013). The starting allele frequency and dominance of a beneficial allele can influence the speed of the sweep and therefore the difference in the trajectory through time from neutrally evolving loci and the required density of sampling points through time needed to detect the sweep (Feder et al. 2014; Malaspinas 2016; Fig. 3). The difficulties in inferring the mode of evolution is nicely illustrated by the re‐evaluation of the trajectory of alleles in genes associated with coat color in horses, which were inferred to have changed consistent with directional selection (Ludwig et al. 2009), drift (Malaspinas 2012), and balancing selection (Steinrücken et al. 2014).

## Detecting Polygenic Selection on a Polygenic Trait

In contrast to phenotypes with a relatively simple genetic basis, polygenic traits are genetically more complex, being determined by the effect of allele frequency changes at hundreds or thousands of loci. Polygenic selection from standing variation might be of equal or greater importance than selective sweeps in rapid adaptive events (Mather 1943; Pritchard and Di Rienzo 2010; Jain and Stephan 2017). Indeed, as many phenotypic traits are polygenic, the quantitative variation associated with these traits is likely to play an important role in adaptation and contribute toward individual fitness in a given set of environmental conditions (Gratten et al. 2008; Besnier et al. 2015; Bosse et al. 2017). Even though the collective effect of polygenic traits under selection can be significant, individual allele frequency shifts are more subtle than those under a selective sweep model, and therefore harder to detect with traditional methods for selection inference. Studies looking for polygenic adaptation in contemporary genomic datasets typically rely on sets of loci associated with a specific trait and identified by genome‐wide association studies (GWAS) (Turchin et al. 2012; Berg and Coop 2014; Robinson et al. 2015; Racimo et al. 2018). Derivations in the mean effect size of a set of loci compared to a null model or another population are indicative of selection. A key limitation of investigating polygenic adaptation using only contemporary samples is in determining the timing and onset of polygenic selection.

Estimation of the timing of polygenic adaptation in an ancestral population can be achieved using just modern samples, but this requires a dataset of known quantitative trait loci and the establishment of the past splits and migration among populations (Racimo et al. 2018). The relationship among populations using population genomic data is increasingly estimated as an admixture graph (Patterson et al. 2012; Pickrell and Pritchard 2012; Lipson et al. 2013). Admixture graphs represent a consensus topology inferred from the majority of neutral loci, in which drift is represented by branch length. Admixture events are then inferred from loci that are a poor fit for this consensus tree model and are incorporated into the graph to increase the fit of the graph to the data. Racimo et al. (2018) expanded this approach to generate an admixture graph from putatively neutral loci. They then separately considered the fit of allele frequency shifts at GWAS loci to the admixture graph to identify when GWAS loci evolved differently to neutral loci (i.e., inconsistent with genetic drift), thereby inferring when polygenic selection occurred in the evolutionary history of the sampled populations. Racimo et al. (2018) proposed that the method should be applicable to admixture graphs that include ancient populations, as commonly incorporated into human population genomic studies (e.g., Lazaridis et al. 2014; Raghavan et al. 2015). However, care is needed, as the method requires sufficient samples from each time period to ensure accurate estimates of allele frequencies and also to avoid artefacts from postmortem damage and low coverage. In addition, linkage disequilibrium structure may vary among populations and through time affecting the accuracy of comparing markers discovered in another population or across temporally stratified data (see Martin et al. 2017). Similar to datasets containing only modern samples, this approach is restricted to species with known GWAS identified loci. A modified version of the *Q_B_* statistic (Racimo et al. 2018), the *S_B_* statistic developed by Refoyo‐Martínez et al. (2019), similarly uses the signal of allele frequency differences between populations, discordant with the consensus topology of an admixture graph. This method does not require gene‐trait association data, making it a promising approach for identifying genome‐wide targets and the timing of selective sweeps in model and nonmodel organisms (Refoyo‐Martínez et al. 2019), but it is unclear if the method would be sufficiently sensitive to detect polygenic selection.

The detection of polygenic selection from time series genetic data requires methods that consider genome‐wide patterns of subtle changes in allele frequencies that are distinguishable from genetic drift. Although the method of Racimo et al. (2018) is dependent upon the loci under selection being known a priori, a theoretical framework developed by Buffalo and Coop (2019) can partition the variance of genome‐wide allele frequency changes through time into those evolving neutrally through drift and those linked to (unknown) loci evolving under additive polygenic selection. However, this approach is subject to many of the caveats discussed below in that it assumes a constant population size and the model would be violated by migration or other temporal variation in population composition. Therefore, although this approach is supported by simulations, and has been demonstrated to be effective at estimating temporal covariance in allele frequencies associated with linked selection in lab‐based experimental evolution (Buffalo and Coop 2019a, 2019b), it may be limited in its application to real‐life aDNA data. The effect of population stratification on polygenic signals from modern samples has recently been highlighted, when two studies found that the signal for height selection in Europe was less pronounced in the U.K. Biobank dataset, which is less confounded by population structure than the GIANT consortium dataset (Berg et al. 2018; Sohail et al. 2019). Sampling through time increases the chances of stratification in a population genomics dataset (Pickrell and Reich 2014), and so would need to be carefully accounted for.

A recent modelling study by Hayward and Sella (2019) found that shifts in mean phenotype toward a new optimum through polygenic adaptation following a sudden environmental change were driven in the short term by the small frequency changes in moderate and large effect alleles. In the long term, the contribution of subtle changes in large‐effect alleles is replaced by large allele frequency changes, including fixation, of moderate and small effect alleles (Hayward and Sella 2019). The ability of temporal sampling approaches, such as those of Racimo et al. (2018) and Buffalo and Coop (2019), may vary between these proposed short‐ and long‐term phases, with the more extreme frequency shifts of the latter intuitively being more detectable. We look forward to future investigations into this temporal change in the signature of polygenic selection.

The results of scans for alleles or genes evolving under polygenic selection can be used to search subnetworks of interacting genes in biological pathways and identify those with unusual features to better understand the interaction with phenotype or the environment. For example, Gouy et al. (2017) applied such a method to identify the polygenic basis and the biological processes involved in convergent adaptation to high altitude in modern humans. The method has been tested on the time series data from Mathieson et al. (2015) and can therefore be applied to aDNA datasets, provided there are sufficient sample sizes, and considering the caveats of population stratification, migration, and linkage disequilibrium changes through time (A. Gouy, pers. comm.). An advantage of incorporating aDNA time series data into such an analysis would be to better determine if selection acts independently at different times on different genes or simultaneously on multiple genes within a network in response to a novel selection pressure: independent and epistatic selection sensu Gouy and Excoffier (2019).

## Detecting Purifying Selection

Negative or purifying selection—the removal of deleterious alleles from a population—can lead to a reduction in genetic diversity in regions of the genome because neutral polymorphisms at sites linked to deleterious mutations are also removed from the population: a process called background selection (Charlesworth et al. 1993). The effectiveness of purifying selection in removing deleterious mutations depends both upon the selection coefficient of the mutation (*s*) and effective population size (*N*_e_), and in an idealized population is thus determined by *N*_e_ *s* (Charlesworth 2009). In this context, we refer to the variance *N*_e_ rather than the inbreeding *N*_e_, the former being the measure of variance in allele frequency drift per generation in an idealized Wright‐Fisher population (Wright 1931; Crow and Denniston 1988). Therefore, although selection will act rapidly to remove strongly deleterious mutations, given a sufficiently large effective population size, weakly deleterious mutations may segregate for multiple generations before they are effectively removed from a population (Kimura et al. 1963). In particular, weakly deleterious recessive mutations can be retained as effectively neutral alleles in heterozygous state (Peischl and Excoffier 2015). The term mutation load is broadly used for the measure of deleterious mutations within an individual (Henn et al. 2015). Prolonged bottlenecks and subsequent expansions, as, for example, under serial founder effects associated with range expansion or domestication events, result in reduced efficacy of selection and increased drift (Lohmueller 2014). As a consequence of these demographic scenarios, weakly deleterious mutations can rise to high frequency within an affected population, and weakly deleterious recessive mutations can become exposed in homozygous form as they rise to fixation through drift; thus under a recessive model, the mutation load resulting from nonequilibrium demography is predicted to have a greater population‐level impact (Peischl and Excoffier 2015). As a result, the signature of background selection detected in comparisons among modern populations can be similar to that of positive polygenic selection in that it reduces genetic diversity and increases genetic differentiation among populations (Charlesworth et al. 1993). Studies solely based on modern population data also lack resolution of the timing of purifying selection relative to demographic changes, for example, pre‐ and post‐bottleneck, when recessive alleles are exposed in homozygous genotypes, or during other demographic events such as extinctions.

In contrast to the methods for detecting positive selection on single or few loci of large effect, which have potentially prohibitively dense temporal sampling requirements for most aDNA datasets currently available, an assessment of the strength of negative selection can be made from a large number of independent (unlinked) loci using relatively few samples. The difficulty is how to disentangle the effects of negative selection from those caused purely by demography, given that both reduce genetic diversity. One approach is to look for differences in genetic diversity across regions of the genome that differ in recombination rate, because the impact of selection on genetic diversity will be greatest where recombination rates are lowest. This approach was used by Murray et al. (2017) in their analysis of DNA from museum samples of the once abundant but now extinct passenger pigeon (*Ectopistes migratorius*). Hung et al. (2014) had previously reported surprisingly low genetic diversity in passenger pigeons and had concluded that this reflected a history of dramatic population size fluctuations. To distinguish between the effects of selection and demography, Murray et al. (2017) mapped their passenger pigeon scaffolds to the chicken genome assembly, and because karyotype and synteny are strongly conserved across bird genomes, they were able to establish that genetic diversity was much lower in regions of the genome with lower rates of recombination. They concluded from this that the much lower than expected genetic diversity of the passenger pigeon was largely a consequence of the impact of selection on linked loci, rather than demographic instability, and they suggested that this might have been a consequence of passenger pigeons having had a very large effective population size.

Although the genomic investigation of the extinct passenger pigeon sampled across a narrow temporal window, other studies have sampled the genomic signature of the extinction process over longer timescales. For example, a loss of genetic diversity and increase in the fraction of the genome composed of runs of homozygosity and accumulation of deleterious mutations were detected in one of the last surviving mammoths (*Mammuthus primigenius*), dated to 4300 years ago, when compared with an older 44,800 years ago sample (Palkopoulou et al. 2015; Rogers and Slatkin 2017). When species recover from a bottleneck rather than going extinct, aDNA time series data can shed light on the strength of purifying selection acting upon the accumulated deleterious mutations. In an ongoing study, a comparison of the per‐genome accumulation of nonsynonymous mutations (see Do et al. 2015) across a global dataset of killer whales (see Foote et al. 2019), the strongest signature of purifying selection is in genomes sampled from Iceland and Norway. Comparing with the genome of a Danish sample dated to 7500 years ago, which was inferred to be ancestral to the modern Icelandic and Norwegian populations, revealed that most of the purging of nonsynonymous mutations had occurred during the Holocene, subsequent to the inferred bottleneck during the last glacial period (see Foote et al. 2016). Thus, as with other forms of selection, sampling across different time periods can inform us of the timing of purifying selection and relate this to changes in demography or environmental variables.

## Detecting Balancing Selection

Balancing selection is the umbrella term used for evolutionary processes that maintain polymorphisms in a population. Different mechanisms can lead to balancing selection. Heterozygote advantage refers to the process whereby individuals with a heterozygous genotype have a higher fitness than those with either homozygous genotype (Lindtke et al. 2017). Under negative frequency dependence, the fitness of a genotype is determined by the frequency of other genotypes, meaning that a genotype remains advantageous if rare. This type of selection is most often found in host‐pathogen or predator‐prey systems (Stahl et al. 1999; Leffler et al. 2013; Le Rouzic et al. 2015; Sato 2018). In genetically structured populations with gene flow, variable selection pressures can result in balancing selection (Levene 1953; Hedrick 2006). Although positive and negative selection have both been extensively studied, balancing selection has gained relatively little attention, likely because it is more difficult to detect as its effects span shorter genomic regions and may be transient in time (Fijarczyk and Babik 2015). As a result, there is little consensus on how prevalent this form of selection is and what role it has in maintaining genetic diversity.

Depending on the time scale, balancing selection will leave different patterns in the genome. Recent balancing selection is characterized by the increase in frequency of an allele at a specific locus. Balancing selection over long evolutionary time scales, on the other hand, will result in increased sequence diversity around the selected locus and long gene genealogies (Charlesworth 2006; Fijarczyk and Babik 2015). However, detecting the footprints of balancing selection in contemporary genomes is not a straightforward task: the patterns left in the genome can either be misinterpreted as other selection processes or may be caused by demography, introgression, or population structure (Fijarczyk and Babik 2015). For example, the signatures of recent or transient balancing selection can be misidentified as ongoing positive selection. Alternatively, signatures from long‐term balancing selection, that is, increased gene diversity, can also be caused by population structure. Due to these difficulties, methods using only contemporary data to detect balancing selection typically have low statistical power.

**Figure 4:**
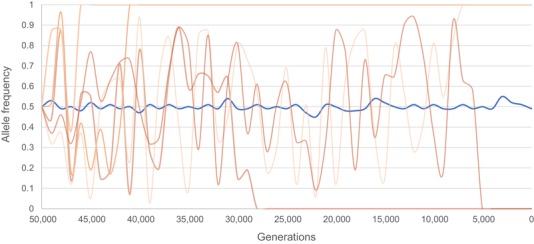
Illustrative scheme of differences in allele frequencies of an allele under balancing selection versus a neutral allele. An allele under balancing selection (shown in blue) will show small fluctuations around a 0.5 allele frequency. The frequencies of neutral alleles (shown in orange) will change following a more stochastic process, eventually leading to fixation or loss of the allele from the population.

Because the frequency of alleles evolving under balancing selection is expected to change less over time than expected under neutral drift (Fig. 4), temporally sampled data can be helpful to detect balancing selection. If alleles are truly under balancing selection, one can expect them to neither reach fixation nor get lost from the population. Although the maintenance of polymorphism is challenging to detect from single‐time point data, these patterns should be detectable over longer periods of time, provided evenly distributed temporal sampling (Fig. 4).

## Caveats and Considerations

The inference of selection of time series paleogenomic data that we have advocated above typically depends upon simple evolutionary models, such as the Wright‐Fisher model, that have a number of assumptions based around an idealized population. In reality, time series aDNA data from most species contravene such models through a history of admixture, overlapping generations and changes in effective population size. Thus, genetic differentiation between temporal samples may be due to drift, selection, or migration (Skoglund et al. 2014). Sample‐rich paleogenomic datasets such as those for horses and humans (Reich 2018; Fages et al. 2019) highlight the fluidity of population structure through time, such that a time series of samples from a given location rarely represents a single continuous population, in which older samples are directly ancestral to younger ones. Furthermore, there can be behavioral differences that can cause sample bias of a subset of a population to accrue in a location (e.g., Allentoft et al. 2010; Pečnerová et al. 2017), thereby invalidating the model assumptions. Ascertainment bias can also occur during the collection of specimens, causing museum datasets to be biased toward a particular sex or phenotype (Cooper et al. 2019). As such, great care is required to rule out migration or population replacement when inferring drift or selection as the driver of allele frequencies from time series data. New approaches are increasingly being developed to estimate how direct of an ancestor an ancient sample is to a modern sample, by estimating the drift along the branch from the most recent common ancestor to the ancient sample (Rasmussen et al. 2014; Racimo et al. 2016; Schlebusch et al. 2017; Schraiber 2018). The shorter the branch, the more directly ancestral the ancient sample was to the modern sample, and thus, the more the dataset represents a continuous population through time. Alternatively, it is possible to test for continuity explicitly using coalescent simulation methods (Bramanti et al. 2009), an approach that was recently extended to include structured populations (Silva et al. 2017). However, a comprehensive investigation into how these confounding variables violate the assumptions and impact the inferences of the methods outlined above is currently lacking. As a minimal next step, further simulation work is needed to understand how migration from sampled or unsampled populations into the study population part way through a time series influences the results. Beyond this, theoretical work is needed to feed into method and tool development that can infer natural selection in datasets with complex demographic histories that violate the assumptions of Wright‐Fisher or other simple models. Additionally, we need a better understanding of the spatial and temporal sampling requirements to be able to detect different types of selection. We have alluded to the fact that changes in processes that can leave a genome‐wide signature, such as polygenic or background selection, can be inferred from even a small number of genomes sampled at a few temporal intervals. In contrast, the temporal signature of processes such as balancing and positive selection on a monogenic or oligogenic trait would require more dense temporal sampling of multiple genomes from each time point to be able to estimate allele frequency variation. However, the effect of the density of temporal and spatial sampling and the number of genomes per sample are additional variables that need to be incorporated into simulations to be able to provide more quantitative and formal guidance for future empirical studies.

## Future Directions

As we enter the futuristic sounding year 2020, a number of methodological and technical advances loom on the near horizon, which we see greatly contributing to the kinds of analyses we have outlined here. Of key importance is the development of methods to handle “big data” such as the genomic datasets composed of hundreds and thousands of individuals (e.g., Reich 2018; Fages et al. 2019). Two recent papers published back‐to‐back (Kelleher et al. 2019; Speidel et al. 2019) together with an accompanying perspective (Harris 2019) introduce new methods, *relate* and *tsinfer*, which estimate genealogies in the presence of recombination at an unprecedented scale. Recombination events result in small differences in the genealogy of contiguous sequences; *tsinfer* records these differences thereby efficiently encoding variation across the genomes of thousands of individuals. This method greatly reduces the data storage requirements and processing time of large datasets of thousands of genomes (Kelleher et al. 2019). The extension of methods such as *tsinfer* and *relate* to aDNA datasets presents new challenges, for example, accounting for postmortem damage patterns and high sequencing error rates, when estimating recombination events. Trees inferred using *tsinfer* have already been used to inform analysis of aDNA (Scheib et al. 2019), and improved methods to deal with the complexities of aDNA are under active development (J Kelleher, pers. comm.).

To accompany these new approaches to encoding the genomic variation within large datasets, machine learning approaches are emerging as valid inferential tool in population genomics (Schrider and Kern 2018; Mondal et al. 2019). Such data‐driven approaches base their inferences by learning the relationship between inputs (e.g., summary statistics of genetic diversity or full genotype information) and outputs (e.g., strength and time of selection) from a large collection of data points, which can be provided by simulations (Sheehan and Song 2016). Machine learning, specifically deep learning and convolutional neural networks, have been successfully applied to population genetic data to infer population size changes (Flagel et al. 2018; Chan et al. 2018) and predict targets of natural selection (Torada et al. 2019). Existing methods can be extended to analyze aDNA data by incorporating (i) the temporal dimension and (ii) missingness of sequencing data obtained from degraded ancient samples. These functionalities can be addressed by employing recurrent layers in the network and encoding the statistical uncertainty of aDNA data in input nodes. As such, deep learning is likely to be a suitable framework for the inference of past selective events from aDNA.

## Summary

Our goal in writing this review was to highlight the potential for paleogenomic time series datasets to enhance our understanding of selective processes, while at the same time cautioning on the many potential pitfalls inherent in working with such challenging samples and datasets. The growth of the field of paleogenomics during the past decade has been close to exponential, and datasets of hundreds of ancient genomes are now available for some study systems. However, it is important to recognize that datasets of this magnitude are still exceptional. Our take on the current state of the field is that the method development for working with paleogenomic datasets has progressed ahead of the widespread availability of such data. But in the knowledge that the generation of many large‐scale datasets for a range of taxa is underway, we anticipate that the relationship between method development and study systems with which to apply them will soon change. We therefore hope that this review will serve to enthuse evolutionary biologists to consider incorporating paleogenomic data in their future study design.

Associate Editor: Z. Gompert

## References

[evl3165-bib-0002] Allentoft, M. E. , M. Bunce , R. P. Scofield , M. L. Hale , and R. N. Holdaway . 2010 Highly skewed sex ratios and biased fossil deposition of moa: ancient DNA provides new insight on New Zealand's extinct megafauna. Quat. Sci. Rev. 29:753–762.

[evl3165-bib-0003] Allentoft, M. E. , M. Collins , D. Harker , J. Haile , C. L. Oskam , M. L. Hale , et al. 2012 The half‐life of DNA in bone: measuring decay kinetics in 158 dated fossils. Proc. R. Soc. B: Biol. Sci. 279:4724–4733. 10.1098/rspb.2012.1745 PMC3497090 23055061

[evl3165-bib-0004] Baldwin‐Brown, J. G. , A. D. Long , and K. R. Thornton . 2014 The power to detect quantitative trait loci using resequenced, experimentally evolved populations of diploid, sexual organisms. Mol. Biol. Evol. 31:1040–1055. 24441104 10.1093/molbev/msu048 PMC3969567

[evl3165-bib-0005] Bank, C. , G. B. Ewing , A. Ferrer‐Admettla , M. Foll , and J. D. Jensen . 2014 Thinking too positive? Revisiting current methods of population genetic selection inference. Trends Genet. 30:540–546. 25438719 10.1016/j.tig.2014.09.010

[evl3165-bib-0006] Barrett, R. D. H. , and D. Schluter . 2008 Adaptation from standing genetic variation. Trends Ecol. Evol. 23:38–44. 18006185 10.1016/j.tree.2007.09.008

[evl3165-bib-0007] Baym, M. , T. D. Lieberman , E. D. Kelsic , R. Chait , R. Gross , I. Yelin , et al. 2016 Spatiotemporal microbial evolution on antibiotic landscapes. Science 353:1147–1151. 27609891 10.1126/science.aag0822 PMC5534434

[evl3165-bib-0008] Beaumont, M. A. 2005 Adaptation and speciation: what can *F_st_* tell us? Trends Ecol. Evol. 20:435–440. 16701414 10.1016/j.tree.2005.05.017

[evl3165-bib-0009] Bennett, A. F. , K. M. Dao , and R. E. Lenski . 1990 Rapid evolution in response to high‐temperature selection. Nature 346:79–81. 2195353 10.1038/346079a0

[evl3165-bib-0010] Berg, J. J. , and G. Coop . 2014 A population genetic signal of polygenic adaptation. PLoS Genet. 10:e1004412. 25102153 10.1371/journal.pgen.1004412 PMC4125079

[evl3165-bib-0011] Berg, J. J. , A. Harpak , N. Sinnott‐Armstrong , A. M. Joergensen , H. Mostafavi , Y. Field , et al. 2019 Reduced signal for polygenic adaptation of height in UK Biobank. eLife 8:e39725. 30895923 10.7554/eLife.39725 PMC6428572

[evl3165-bib-0012] Besnier, F. , K. A. Glover , S. Lien , M. Kent , M. M. Hansen , X. Shen , et al. 2015 Identification of quantitative genetic components of fitness variation in farmed, hybrid and native salmon in the wild. Heredity 115:47–55. 26059968 10.1038/hdy.2015.15 PMC4815496

[evl3165-bib-0013] Bollback, J. P. , T. L. York , and R. Nielsen . 2008 Estimation of 2Nes from temporal allele frequency data. Genetics 179:497–502. 18493066 10.1534/genetics.107.085019 PMC2390626

[evl3165-bib-0014] Bosse, M. , L. G. Spurgin , V. N. Laine , E. F. Cole , J. A. Firth , P. Gienapp , et al. 2017 Recent natural selection causes adaptive evolution of an avian polygenic trait. Science 358:365–368. 29051380 10.1126/science.aal3298

[evl3165-bib-0015] Bosshard, L. , I. Dupanloup , O. Tenaillon , R. Bruggmann , M. Ackermann , S. Peischl , et al. 2017 Accumulation of deleterious mutations during bacterial range expansions. Genetics 207:669–684. 28821588 10.1534/genetics.117.300144 PMC5629331

[evl3165-bib-0016] Bramanti, B. , M. G. Thomas , W. Haak , M. Unterlaender , P. Jores , K. Tambets , et al. 2009 Genetic discontinuity between local hunter‐gatherers and central Europe's first farmers. Science 326:137–140. 19729620 10.1126/science.1176869

[evl3165-bib-0018] Brunson, K. , and D. Reich . 2019 The promise of paleogenomics beyond our own species. Trends Genet. 35:319–329. 30954285 10.1016/j.tig.2019.02.006

[evl3165-bib-0019] Buckley, M. T. , F. Racimo , M. E. Allentoft , M. K. Jensen , A. Jonsson , H. Huang , et al. 2017 Selection in Europeans on fatty acid desaturases associated with dietary changes. Mol. Biol. Evol. 34:1307–1318. 28333262 10.1093/molbev/msx103 PMC5435082

[evl3165-bib-0020] Buffalo, V. , and G. Coop . 2019a The linked selection signature of rapid adaptation in temporal genomic data. Genetics. 10.1534/genetics.119.302581. PMC6827383 31558582

[evl3165-bib-0021] Buffalo, V. , and G. Coop . 2019b Estimating the genome‐wide contribution of selection to temporal allele frequency change. BioRxiv. 10.1101/798595. PMC7456072 32817464

[evl3165-bib-0022] Burri, R. , A. Nater , T. Kawakami , C. F. Mugal , P. I. Olason , L. Smeds , et al. 2015 Linked selection and recombination rate variation drive the evolution of the genomic landscape of differentiation across the speciation continuum of *Ficedula* flycatchers. Genome Res. 25:1656–1665. 26355005 10.1101/gr.196485.115 PMC4617962

[evl3165-bib-0023] Burri, R. 2017 Interpreting differentiation landscapes in the light of long‐term linked selection. Evol. Lett. 1:118–131.

[evl3165-bib-0024] Carøe, C. , S. Gopalakrishnan , L. Vinner , S. S. Mak , M. H. S. Sinding , J. A. Samaniego , et al. 2018 Single‐tube library preparation for degraded DNA. Methods Ecol. Evol. 9:410–419.

[evl3165-bib-0025] Chan, J. , V. Perrone , J. Spence , P. Jenkins , S. Mathieson , and Y. Song . 2018 A likelihood‐free inference framework for population genetic data using exchangeable neural networks. In: Advances in neural information processing systems, 8594–8605. PMC7687905 33244210

[evl3165-bib-0026] Charlesworth, D. 2006 Balancing selection and its effects on sequences in nearby genome regions. PLoS Genet. 2:379–384. 10.1371/journal.pgen.0020064 PMC1449905 16683038

[evl3165-bib-0027] Charlesworth, B. 2009 Effective population size and patterns of molecular evolution and variation. Nature Rev. Genet. 10:195–205. 19204717 10.1038/nrg2526

[evl3165-bib-0029] Charlesworth, B. , M. T. Morgan , and D. Charlesworth . 1993 The effect of deleterious mutations on neutral molecular variation. Genetics 134:1289–1303. 8375663 10.1093/genetics/134.4.1289 PMC1205596

[evl3165-bib-0031] Comeron, J. M. 2014 Background selection as baseline for nucleotide variation across the *Drosophila* genome. PLoS Genet. 10:e1004434. 24968283 10.1371/journal.pgen.1004434 PMC4072542

[evl3165-bib-0032] Cooper, N. , A. L. Bond , J. L. Davis , R. Portela Miguez , L. Tomsett , and K. M. Helgen . 2019 Sex biases in bird and mammal natural history collections. Proc. R. Soc. B 286:2019–2025. 10.1098/rspb.2019.2025 PMC6834056 31640514

[evl3165-bib-0034] Crisci, J. L. , Y. P. Poh , A. Bean , A. Simkin , and J. D. Jensen . 2012 Recent progress in polymorphism‐based population genetic inference. J. Hered. 103:287–296. 22246406 10.1093/jhered/esr128

[evl3165-bib-0035] Crow, J. F. , and C. Denniston . 1988 Inbreeding and variance effective population numbers. Evolution 42:482–495. 28564013 10.1111/j.1558-5646.1988.tb04154.x

[evl3165-bib-0036] Cruickshank, T. E. , and M. W. Hahn . 2014 Reanalysis suggests that genomic islands of speciation are due to reduced diversity, not reduced gene flow. Mol. Ecol. 23:3133–3157. 24845075 10.1111/mec.12796

[evl3165-bib-0037] Dabney, J. , M. Knapp , I. Glocke , M. T. Gansauge , A. Weihmann , B. Nickel , et al. 2013 Complete mitochondrial genome sequence of a Middle Pleistocene cave bear reconstructed from ultrashort DNA fragments. Proc. Natl. Acad. Sci. 110:15758–15763. 24019490 10.1073/pnas.1314445110 PMC3785785

[evl3165-bib-0038] Dabney, J. , and M. Meyer . 2019 Extraction of highly degraded DNA from ancient bones and teeth Pp. 25–29 *in* Shapiro B., Barlow A., Heintzman P. D., Hofreiter M., Paijmans J. L. A., and Soares A. E. R., eds.Ancient DNA. Humana Press, New York. 10.1007/978-1-4939-9176-1_4 30875041

[evl3165-bib-0041] Do, R. , D. Balick , H. Li , I. Adzhubei , S. Sunyaev , and D. Reich . 2015 No evidence that selection has been less effective at removing deleterious mutations in Europeans than in Africans. Nat. Genet. 47:126–131. 25581429 10.1038/ng.3186 PMC4310772

[evl3165-bib-0053] da Fonseca, R. R. , B. D. Smith , N. Wales , E. Cappellini , P. Skoglund , M. Fumagalli , et al. 2015 The origin and evolution of maize in the Southwestern United States. Nat. Plants 1:14003. 27246050 10.1038/nplants.2014.3

[evl3165-bib-0042] Epstein, B. , M. Jones , R. Hamede , S. Hendricks , H. McCallum , E. P. Murchison , et al. 2016 Rapid evolutionary response to a transmissible cancer in Tasmanian devils. Nat. Commun. 7:12684. 27575253 10.1038/ncomms12684 PMC5013612

[evl3165-bib-0043] Excoffier, L. , M. Foll , and R. J. Petit . 2009 Genetic consequences of range expansions. Annu. Rev. Ecol. Evol. Syst. 40:481–501.

[evl3165-bib-0044] Fages, A. , K. Hanghøj , N. Khan , C. Gaunitz , A. Seguin‐Orlando , M. Leonardi , et al. 2019 Tracking five millennia of horse management with extensive ancient genome time series. Cell 177:1419–1435. 31056281 10.1016/j.cell.2019.03.049 PMC6547883

[evl3165-bib-0045] Fang, B. , P. Kemppainen , P. Momigliano , and J. Merilä . 2019 Oceans apart: Heterogeneous patterns of parallel evolution in sticklebacks. BioRxiv. 10.1101/826412.

[evl3165-bib-0046] Fay, J. C. , and C. I. Wu . 2000 Hitchhiking under positive Darwinian selection. Genetics 155:1405–1413. 10880498 10.1093/genetics/155.3.1405 PMC1461156

[evl3165-bib-0047] Feder, J. L. , S. P. Egan , and P. Nosil . 2012 The genomics of speciation‐with‐gene‐flow. Trends Genet. 28:342–350. 22520730 10.1016/j.tig.2012.03.009

[evl3165-bib-0178] Feder, A. F. , S. Kryazhimskiy , and J. B. Plotkin . 2014 Identifying signatures of selection in genetic time series. Genetics 196:509–522. 24318534 10.1534/genetics.113.158220 PMC3914623

[evl3165-bib-0048] Ferrer‐Admetlla, A. , C. Leuenberger , J. D. Jensen , and D. Wegmann . 2016 An approximate Markov model for the Wright–Fisher diffusion and its application to time series data. Genetics 203:831–846. 27038112 10.1534/genetics.115.184598 PMC4896197

[evl3165-bib-0049] Fijarczyk, A. , and W. Babik . 2015 Detecting balancing selection in genomes: limits and prospects. Mol. Ecol. 24:3529–3545. 25943689 10.1111/mec.13226

[evl3165-bib-0050] Fisher, R. A. , and E. B. Ford . 1947 The spread of a gene in natural conditions in a colony of the moth *Panaxia dominula* L. Heredity 1:143–174.

[evl3165-bib-0051] Flagel, L. , Y. Brandvain , and D. R. Schrider . 2018 The unreasonable effectiveness of convolutional neural networks in population genetic inference. Mol. Biol. Evol. 36:220–238. 10.1093/molbev/msy224 PMC6367976 30517664

[evl3165-bib-0052] Foll, M. , H. Shim , and J. D. Jensen . 2015 WFABC: a Wright–Fisher ABC‐based approach for inferring effective population sizes and selection coefficients from time‐sampled data. Mol. Ecol. Res. 15:87–98. 10.1111/1755-0998.12280 24834845

[evl3165-bib-0054] Foote, A. D. , N. Vijay , M. C. Ávila‐Arcos , R. W. Baird , J. W. Durban , M. Fumagalli , et al. 2016 Genome‐culture coevolution promotes rapid divergence of killer whale ecotypes. Nature Comm. 7:11693. 10.1038/ncomms11693 PMC4895049 27243207

[evl3165-bib-0055] Foote, A. D. , M. D. Martin , M. Louis , G. Pacheco , K. M. Robertson , M.‐H. S. Sinding , et al. 2019 Killer whale genomes reveal a complex history of recurrent admixture and vicariance. Mol. Ecol. 28:3427–3444. 31131963 10.1111/mec.15099

[evl3165-bib-0056] Franks, S. J. , N. C. Kane , N. B. O'Hara , S. Tittes , and J. S. Rest . 2016 Rapid genome‐wide evolution in *Brassica rapa* populations following drought revealed by sequencing of ancestral and descendant gene pools. Mol. Ecol. 25:3622–3631. 27072809 10.1111/mec.13615 PMC4963267

[evl3165-bib-0057] Gansauge, M. T. , T. Gerber , I. Glocke , P. Korlević , L. Lippik , S. Nagel , et al. 2017 Single‐stranded DNA library preparation from highly degraded DNA using T4 DNA ligase. Nucleic Acids Res. 45:e79. 28119419 10.1093/nar/gkx033 PMC5449542

[evl3165-bib-0058] Gompert, Z. 2016 Bayesian inference of selection in a heterogeneous environment from genetic time‐series data. Mol. Ecol. 25:121–134. 26184577 10.1111/mec.13323

[evl3165-bib-0059] Good, B. H. , M. J. McDonald , J. E. Barrick , R. E. Lenski , and M. M. Desai . 2017 The dynamics of molecular evolution over 60,000 generations. Nature 551:45–50. 29045390 10.1038/nature24287 PMC5788700

[evl3165-bib-0060] Gouy, A. , J. T. Daub , and L. Excoffier . 2017 Detecting gene subnetworks under selection in biological pathways. Nucleic Acids Res. 45:e149. 28934485 10.1093/nar/gkx626 PMC5766194

[evl3165-bib-0061] Gouy, A. , and L. Excoffier . 2019 Polygenic patterns of adaptive introgression in modern humans are mainly shaped by response to pathogens. BioRxiv. 10.1101/732958. 31935281

[evl3165-bib-0062] Grant, P. R. , and R. B. Grant . 2002 Unpredictable evolution in a 30 year study of Darwin's finches. Science 296:707–711. 11976447 10.1126/science.1070315

[evl3165-bib-0063] Gratten, J. , A. J. Wilson , A. F. McRae , D. Beraldi , P. M. Visscher , J. M. Pemberton , et al. 2008 A localized negative genetic correlation constrains microevolution of coat color in wild sheep. Science 319:318–320. 18202287 10.1126/science.1151182

[evl3165-bib-0065] Harris, K. 2019 From a database of genomes to a forest of evolutionary trees. Nature Genet. 51:1306–1307. 31477932 10.1038/s41588-019-0492-x PMC8195310

[evl3165-bib-0066] Hayward, L. K. , and G. Sella . 2019 Polygenic adaptation after a sudden change in environment. BioRxiv. 10.1101/792952. PMC9683794 36155653

[evl3165-bib-0067] Hedrick, P. W. 2006 Genetic polymorphism in heterogeneous environments: the age of genomics. Annu. Rev. Ecol. Evol. Syst. 37:67–93.

[evl3165-bib-0069] Hendry, A. P. , J. K. Wenburg , P. Bentzen , E. C. Volk , and T. P. Quinn . 2000 Rapid evolution of reproductive isolation in the wild: evidence from introduced salmon. Science 290:516–518. 11039932 10.1126/science.290.5491.516

[evl3165-bib-0070] Henn, B. M. , L. R. Botigué , C. D. Bustamante , A. G. Clark , and S. Gravel . 2015 Estimating the mutation load in human genomes. Nature Rev. Genet. 16:333–343. 25963372 10.1038/nrg3931 PMC4959039

[evl3165-bib-0071] Hermisson, J. , and P. S. Pennings . 2017 Soft sweeps and beyond: understanding the patterns and probabilities of selection footprints under rapid adaptation. Methods Ecol. Evol. 8:700–716.

[evl3165-bib-0072] Hung, C. M. , P. J. L. Shaner , R. M. Zink , W. C. Liu , T. C. Chu , et al. 2014 Drastic population fluctuations explain the rapid extinction of the passenger pigeon. Proc. Natl. Acad. Sci. 111:10636–10641. 24979776 10.1073/pnas.1401526111 PMC4115547

[evl3165-bib-0073] Irving‐Pease, E. K. , H. Ryan , A. Jamieson , E. A. Dimopoulos , G. Larson , and L. A. Frantz . 2018 Paleogenomics of animal domestication Pp. 225–272 *in* Lindqvist C. and Rajora O., eds. Paleogenomics. Springer, Cham, Switzerland.

[evl3165-bib-0074] Jain, K. , and W. Stephan . 2017 Rapid adaptation of a polygenic trait after a sudden environmental shift. Genetics 206:389–406. 28341654 10.1534/genetics.116.196972 PMC5419483

[evl3165-bib-0076] Kelleher, J , Y. Wong , A. W. Wohns , C. Fadil , P. K. Albers , and G. McVean . 2019 Inferring whole‐genome histories in large population datasets. Nat. Genet. 51:1330–1338. 31477934 10.1038/s41588-019-0483-y PMC6726478

[evl3165-bib-0077] Kelly, J. K. , and K. A. Hughes . 2019 Pervasive linked selection and intermediate‐frequency alleles are implicated in an evolve‐and‐resequencing experiment of *Drosophila simulans* . Genetics 211:943–961. 30593495 10.1534/genetics.118.301824 PMC6404262

[evl3165-bib-0078] Kim, Y. , and R. Nielsen . 2004 Linkage disequilibrium as a signature of selective sweeps. Genetics 167:1513–1524. 15280259 10.1534/genetics.103.025387 PMC1470945

[evl3165-bib-0079] Kimura, M. , T. Maruyama , and J. F. Crow . 1963 The mutation load in small populations. Genetics 48:1303–1312. 14071753 10.1093/genetics/48.10.1303 PMC1210420

[evl3165-bib-0081] Lazaridis, I. , N. Patterson , A. Mittnik , G. Renaud , S. Mallick , K. Kirsanow , et al. 2014 Ancient human genomes suggest three ancestral populations for present‐day Europeans. Nature 513:409–413. 25230663 10.1038/nature13673 PMC4170574

[evl3165-bib-0082] Leffler, E. M. , Z. Gao , S. Pfeifer , L. Ségurel , A. Auton , O. Venn , et al. 2013 Multiple instances of ancient balancing selection shared between humans and chimpanzees. Science 340:1578–1582. 10.1126/science.1234070 PMC3612375 23413192

[evl3165-bib-0083] Le Rouzic, A. , T. F. Hansen , T. P. Gosden , and E. I. Svensson . 2015 Evolutionary time‐series analysis reveals the signature of frequency‐dependent selection on a female mating polymorphism. Am. Nat. 185:E182–E196. 25996869 10.1086/680982

[evl3165-bib-0084] Levene, H. 1953 Genetic equilibrium when more than one ecological niche is available. Am. Nat. 87:331–333.

[evl3165-bib-0085] Lewontin, R. C. , and J. Krakauer . 1973 Distribution of gene frequency as a test of the theory of the selective neutrality of polymorphisms. Genetics 74:175–195. 4711903 10.1093/genetics/74.1.175 PMC1212935

[evl3165-bib-0086] Li, J. , H. Li , M. Jakobsson , S. Li , P. Sjödin , and M. Lascoux . 2012 Joint analysis of demography and selection in population genetics: where do we stand and where could we go? Mol. Ecol. 21:28–44. 21999307 10.1111/j.1365-294X.2011.05308.x

[evl3165-bib-0087] Librado, P. , C. Gamba , C. Gaunitz , C. Der Sarkissian , M. Pruvost , A. Albrechtsen , et al. 2017 Ancient genomic changes associated with domestication of the horse. Science 356:442–445. 28450643 10.1126/science.aam5298

[evl3165-bib-0088] Lindahl, T. 1993 Instability and decay of the primary structure of DNA. Nature 362:709–715. 8469282 10.1038/362709a0

[evl3165-bib-0089] Lindtke, D. , K. Lucek , V. Soria‐Carrasco , R. Villoutreix , T. E. Farkas , R. Riesch , et al. 2017 Long‐term balancing selection on chromosomal variants associated with crypsis in a stick insect. Mol. Ecol. 26:6189–6205. 28786544 10.1111/mec.14280

[evl3165-bib-0090] Link, V. , A. Kousathanas , K. Veeramah , C. Sell , A. Scheu , and D. Wegmann . 2017 ATLAS: analysis tools for low‐depth and ancient samples. BioRxiv. 10.1101/105346.

[evl3165-bib-0092] Lipson, M. , P. R. Loh , A. Levin , D. Reich , N. Patterson , and B. Berger . 2013 Efficient moment‐based inference of admixture parameters and sources of gene flow. Mol. Biol. Evol. 30:1788–1802. 23709261 10.1093/molbev/mst099 PMC3708505

[evl3165-bib-0093] Lohmueller, K. E. 2014 The distribution of deleterious genetic variation in human populations. Curr. Opin. Genet. Dev. 29:139–146. 25461617 10.1016/j.gde.2014.09.005

[evl3165-bib-0094] Long, A. , G. Liti , A. Luptak , and O. Tenaillon . 2015 Elucidating the molecular architecture of adaptation via evolve and resequence experiments. Nat. Rev. Genet. 16:567–582. 26347030 10.1038/nrg3937 PMC4733663

[evl3165-bib-0095] Ludwig, A. , M. Pruvost , M. Reissmann , N. Benecke , G. A. Brockmann , P. Castaños , et al. 2009 Coat color variation at the beginning of horse domestication. Science 324:485–485. 19390039 10.1126/science.1172750 PMC5102060

[evl3165-bib-0096] Malaspinas, A. S. , O. Malaspinas , S. N. Evans , and M. Slatkin . 2012 Estimating allele age and selection coefficient from time‐serial data. Genetics 192:599–607. 22851647 10.1534/genetics.112.140939 PMC3454883

[evl3165-bib-0097] Malaspinas, A. S. 2016 Methods to characterize selective sweeps using time serial samples: an ancient DNA perspective. Mol. Ecol. 25:24–41. 26613371 10.1111/mec.13492

[evl3165-bib-0099] Marques, D. A. , F. C. Jones , F. Di Palma , D. M. Kingsley , and T. E. Reimchen . 2018 Experimental evidence for rapid genomic adaptation to a new niche in an adaptive radiation. Nat. Ecol. Evol. 2:1128–1138. 29942074 10.1038/s41559-018-0581-8 PMC6519129

[evl3165-bib-0100] Marques, D. A. , J. I. Meier , and O. Seehausen . 2019 A combinatorial view on speciation and adaptive radiation. Trends Ecol. Evol. 34:531–544. 30885412 10.1016/j.tree.2019.02.008

[evl3165-bib-0101] Martiniano, R. , E. Garrison , E. R. Jones , A. Manica , and R. Durbin . 2019 Removing reference bias in ancient DNA data analysis by mapping to a sequence variation graph. BioRxiv. 10.1101/782755. PMC7499850 32943086

[evl3165-bib-0102] Martin, A. R. , C. R. Gignoux , R. K. Walters , G. L. Wojcik , B. M. Neale , S. Gravel , et al. 2017 Human demographic history impacts genetic risk prediction across diverse populations. Am. J. Hum. Genet. 100:635–649. 28366442 10.1016/j.ajhg.2017.03.004 PMC5384097

[evl3165-bib-0103] Martin, S. H. , K. K. Dasmahapatra , N. J. Nadeau , C. Salazar , J. R. Walters , F. Simpson , et al. 2013 Genome‐wide evidence for speciation with gene flow in *Heliconius* butterflies. Genome Res. 23:1817–1828. 24045163 10.1101/gr.159426.113 PMC3814882

[evl3165-bib-0104] Mather, K. 1943 Polygenic inheritance and natural selection. Biol. Rev. 18:32–64.

[evl3165-bib-0105] Mathieson, I. 2019 Limited evidence for selection at the *FADS* locus in Native American populations. BioRxiv. 10.1101/2019.12.11.873356. PMC7306688 32145021

[evl3165-bib-0106] Mathieson, I. , I. Lazaridis , N. Rohland , S. Mallik , N. Patterson , S. A. Roodenberg , et al. 2015 Genome‐wide patterns of selection in 230 ancient Eurasians. Nature 528:499–503. 26595274 10.1038/nature16152 PMC4918750

[evl3165-bib-0107] Mathieson, I. , and G. McVean , 2013 Estimating selection coefficients in spatially structured populations from time series data of allele frequencies. Genetics 193:973–984. 23307902 10.1534/genetics.112.147611 PMC3584010

[evl3165-bib-0108] Mathieson, S. , and I. Mathieson , 2018 FADS1 and the timing of human adaptation to agriculture. Mol. Biol. Evol. 35:2957–2970. 30272210 10.1093/molbev/msy180 PMC6278866

[evl3165-bib-0109] Meyer, M. , J. L. Arsuaga , C. de Filippo , S. Nagel , A. Aximu‐Petri , B. Nickel , et al. 2016 Nuclear DNA sequences from the Middle Pleistocene Sima de los Huesos hominins. Nature 531:504–507. 26976447 10.1038/nature17405

[evl3165-bib-0110] Miller, S. E. , M. Roesti , and D. Schluter . 2019 A single interacting species leads to widespread parallel evolution of the stickleback genome. Curr. Biol. 29:530–537. 30686736 10.1016/j.cub.2018.12.044 PMC6371808

[evl3165-bib-0111] Mondal, M. , J. Bertranpetit , and O. Lao . 2019 Approximate Bayesian computation with deep learning supports a third archaic introgression in Asia and Oceania. Nat. Commun. 10:246. 30651539 10.1038/s41467-018-08089-7 PMC6335398

[evl3165-bib-0112] Murray, G. G. , A. E. Soares , B. J. Novak , N. K. Schaefer , J. A. Cahill , A. J. Baker , et al. 2017 Natural selection shaped the rise and fall of passenger pigeon genomic diversity. Science 358:951–954. 29146814 10.1126/science.aao0960

[evl3165-bib-0113] Navascués, M. , F. Depaulis , and B. C. Emerson . 2010 Combining contemporary and ancient DNA in population genetic and phylogeographical studies. Mol. Ecol. Resour. 10:760–772. 21565088 10.1111/j.1755-0998.2010.02895.x

[evl3165-bib-0114] Nielsen, R. 2005 Molecular signatures of natural selection. Annu. Rev. Genet. 39:197–218. 16285858 10.1146/annurev.genet.39.073003.112420

[evl3165-bib-0117] Ollivier, M. , A. Tresset , F. Bastian , L. Lagoutte , E. Axelsson , M. L. Arendt , et al. 2016 *Amy2B* copy number variation reveals starch diet adaptations in ancient European dogs. R. Soc. open Sci. 3:160449. 28018628 10.1098/rsos.160449 PMC5180126

[evl3165-bib-0118] Orlando, L. , A. Ginolhac , G. Zhang , D. Froese , A. Albrechtsen , M. Stiller , et al. 2013 Recalibrating Equus evolution using the genome sequence of an early Middle Pleistocene horse. Nature 499:74–78. 23803765 10.1038/nature12323

[evl3165-bib-0119] Palkopoulou, E. , S. Mallick , P. Skoglund , J. Enk , N. Rohland , H. Li , et al. 2015 Complete genomes reveal signatures of demographic and genetic declines in the woolly mammoth. Curr. Biol. 25:1395–1400. 25913407 10.1016/j.cub.2015.04.007 PMC4439331

[evl3165-bib-0120] Patterson, N. , P. Moorjani , Y. Luo , S. Mallick , N. Rohland , Y. Zhan , et al. 2012 Ancient admixture in human history. Genetics 192:1065–1093. 22960212 10.1534/genetics.112.145037 PMC3522152

[evl3165-bib-0121] Peischl, S. , and L. Excoffier . 2015 Expansion load: recessive mutations and the role of standing genetic variation. Mol. Ecol. 24:2084–2094. 25786336 10.1111/mec.13154

[evl3165-bib-0122] Pečnerová, P. , D. Díez‐del‐Molino , N. Dussex , T. Feuerborn , J. von Seth , J. van der Plicht , et al. 2017 Genome‐based sexing provides clues about behavior and social structure in the woolly mammoth. Curr. Biol. 27:3505–3510. 29103934 10.1016/j.cub.2017.09.064

[evl3165-bib-0124] Pickrell, J. K. , and J. K. Pritchard . 2012 Inference of population splits and mixtures from genome‐wide allele frequency data. PLoS Genet. 8:e1002967. 23166502 10.1371/journal.pgen.1002967 PMC3499260

[evl3165-bib-0125] Pickrell, J. K. , and D. Reich . 2014 Toward a new history and geography of human genes informed by ancient DNA. Trends Genet. 30:377–389. 25168683 10.1016/j.tig.2014.07.007 PMC4163019

[evl3165-bib-0126] Pinhasi, R. , D. Fernandes , K. Sirak , M. Novak , S. Connell , S. Alpaslan‐Roodenberg , et al. 2015 Optimal ancient DNA yields from the inner ear part of the human petrous bone. PLoS One 10:e0129102. 26086078 10.1371/journal.pone.0129102 PMC4472748

[evl3165-bib-0127] Pouyet, F. , S. Aeschbacher , A. Thiéry , and L. Excoffier . 2018 Background selection and biased gene conversion affect more than 95% of the human genome and bias demographic inferences. Elife 7:e36317. 30125248 10.7554/eLife.36317 PMC6177262

[evl3165-bib-0128] Pritchard, J. K. , and A. Di Rienzo . 2010 Adaptation–not by sweeps alone. Nature Rev. Genet. 11:665–667. 20838407 10.1038/nrg2880 PMC4652788

[evl3165-bib-0129] R Core Team . 2019 R: a language and environment for statistical computing. R Foundation for Statistical Computing, Vienna, Austria.

[evl3165-bib-0130] Racimo, F. , J. J. Berg , and J. K. Pickrell . 2018 Detecting polygenic adaptation in admixture graphs. Genetics 208:1565–1584. 29348143 10.1534/genetics.117.300489 PMC5887149

[evl3165-bib-0131] Racimo, F. , G. Renaud , and M. Slatkin . 2016 Joint estimation of contamination, error and demography for nuclear DNA from ancient humans. PLoS Genet. 12:e1005972. 27049965 10.1371/journal.pgen.1005972 PMC4822957

[evl3165-bib-0132] Raghavan, M. , M. Steinrücken , K. Harris , S. Schiffels , S. Rasmussen , M. DeGiorgio , et al. 2015 Genomic evidence for the Pleistocene and recent population history of Native Americans. Science 349:aab3884. 26198033 10.1126/science.aab3884 PMC4733658

[evl3165-bib-0133] Rajpurohit, S. , E. Gefen , A. O. Bergland , D. A. Petrov , A. G. Gibbs , and P. S. Schmidt . 2018 Spatiotemporal dynamics and genome‐wide association analysis of desiccation tolerance in *Drosophila melanogaster* . Mol. Ecol. 27:3525–3540. 30051644 10.1111/mec.14814 PMC6129450

[evl3165-bib-0134] Ramos‐Madrigal, J. , B. D. Smith , J. V. Moreno‐Mayar , S. Gopalakrishnan , J. Ross‐Ibarra , M. T. P. Gilbert , et al. 2016 Genome sequence of a 5,310‐year‐old maize cob provides insights into the early stages of maize domestication. Curr. Biol. 26:3195–3201. 27866890 10.1016/j.cub.2016.09.036

[evl3165-bib-0135] Rasmussen, M. , S. L. Anzick , M. R. Waters , P. Skoglund , M. DeGiorgio , T. W. Jr. Stafford , et al. 2014 The genome of a Late Pleistocene human from a Clovis burial site in western Montana. Nature 506:225–229. 24522598 10.1038/nature13025 PMC4878442

[evl3165-bib-0136] Reich, D. 2018 Who we are and how we got here: Ancient DNA and the new science of the human past. Oxford Univ. Press, Oxford, U.K.

[evl3165-bib-0137] Refoyo‐Martínez, A. , R. R. da Fonseca , K. Halldórsdóttir , E. Árnason , T. Mailund , and F. Racimo . 2019 Identifying loci under positive selection in complex population histories. Genome Res. 29:1506–1520. 31362936 10.1101/gr.246777.118 PMC6724678

[evl3165-bib-0138] Renaud, G. , M. Schubert , S. Sawyer , and L. Orlando . 2019 Authentication and assessment of contamination in ancient DNA Pp. 163–194 *in* Shapiro B., Barlow A., Heintzman P. D., Hofreiter M., Paijmans J. L. A., and Soares A. E. R., eds.Ancient DNA. Humana Press, New York. 10.1007/978-1-4939-9176-1_17 30875054

[evl3165-bib-0139] Robinson, M. R. , G. Hemani , C. Medina‐Gomez , M. Mezzavilla , T. Esko , K. Shakhbazov , et al. 2015 Population genetic differentiation of height and body mass index across Europe. Nat. Genet 47:1357–1362. 26366552 10.1038/ng.3401 PMC4984852

[evl3165-bib-0140] Rogers, R. L. , and M. Slatkin . 2017 Excess of genomic defects in a woolly mammoth on Wrangel island. PLoS Genet. 13:e1006601. 28253255 10.1371/journal.pgen.1006601 PMC5333797

[evl3165-bib-0142] Sato, Y. 2018 Associational effects and the maintenance of polymorphism in plant defense against herbivores: review and evidence. Plant Species Biol. 33:91–108.

[evl3165-bib-0143] Scheib, C. L. , R. Hui , E. D'Atanasio , A. W. Wohns , S. A. Inskip , A. Rose , et al. 2019 East Anglian early Neolithic monument burial linked to contemporary Megaliths. Ann. Hum. Biol. 46:145‐149. 31184205 10.1080/03014460.2019.1623912 PMC6816495

[evl3165-bib-0144] Schlebusch, C. M. , H. Malmström , T. Günther , P. Sjödin , A. Coutinho , H. Edlund , et al. 2017 Southern African ancient genomes estimate modern human divergence to 350,000 to 260,000 years ago. Science 358:652–655. 28971970 10.1126/science.aao6266

[evl3165-bib-0145] Schlötterer, C. , R. Kofler , E. Versace , R. Tobler , and S. U. Franssen . 2015 Combining experimental evolution with next‐generation sequencing: a powerful tool to study adaptation from standing genetic variation. Heredity 114:431–440. 25269380 10.1038/hdy.2014.86 PMC4815507

[evl3165-bib-0147] Schluter, D. , and G. L. Conte . 2009 Genetics and ecological speciation. Proc. Natl. Acad. Sci. 106:9955–9962. 19528639 10.1073/pnas.0901264106 PMC2702799

[evl3165-bib-0148] Schraiber, J. G. 2018 Assessing the relationship of ancient and modern populations. Genetics 208:383–398. 29167200 10.1534/genetics.117.300448 PMC5753871

[evl3165-bib-0149] Schraiber, J. G. , S. N. Evans , and M. Slatkin . 2016 Bayesian inference of natural selection from allele frequency time series. Genetics 203:493–511. 27010022 10.1534/genetics.116.187278 PMC4858794

[evl3165-bib-0150] Schrider, D. R. , and A. D. Kern . 2018 Supervised machine learning for population genetics: a new paradigm. Trends Genet. 34:301–312. 29331490 10.1016/j.tig.2017.12.005 PMC5905713

[evl3165-bib-0152] Seehausen, O. , R. K. Butlin , I. Keller , C. E. Wagner , J. W. Boughman , P. A. Hohenlohe , et al. 2014 Genomics and the origin of species. Nat. Rev. Genet. 15:176–192. 24535286 10.1038/nrg3644

[evl3165-bib-0154] Shaw, K. L. , and S. P. Mullen . 2014 Speciation continuum. J. Hered. 105:741–742. 10.1093/jhered/esu060 25149250

[evl3165-bib-0155] Sheehan, S. , and Y. S. Song . 2016 Deep learning for population genetic inference. PLoS Comput. Biol. 12:e1004845. 27018908 10.1371/journal.pcbi.1004845 PMC4809617

[evl3165-bib-0156] Shim, H. , S. Laurent , S. Matuszewski , M. Foll , and J. D. Jensen . 2016 Detecting and quantifying changing selection intensities from time‐sampled polymorphism data. G3 6:893–904. 26869618 10.1534/g3.115.023200 PMC4825659

[evl3165-bib-0157] Silva, N. M. , J. Rio , and M. Currat . 2017 Investigating population continuity with ancient DNA under a spatially explicit simulation framework. BMC Genet. 18:114. 29246100 10.1186/s12863-017-0575-6 PMC5731203

[evl3165-bib-0158] Skoglund, P. , and I. Mathieson . 2018 Ancient genomics of modern humans: the first decade. Annu. Rev. Genomics Hum. Genet. 19:381–404. 29709204 10.1146/annurev-genom-083117-021749

[evl3165-bib-0159] Skoglund, P. , P. Sjödin , T. Skoglund , M. Lascoux , and M. Jakobsson . 2014 Investigating population history using temporal genetic differentiation. Mol. Biol. Evol. 31:2516–2527. 24939468 10.1093/molbev/msu192 PMC4137715

[evl3165-bib-0160] Sohail, M. , R. M. Maier , A. Ganna , A. Bloemendal , A. R. Martin , M. C. Turchin , et al. 2019 Polygenic adaptation on height is overestimated due to uncorrected stratification in genome‐wide association studies. eLife 8:e39702. 30895926 10.7554/eLife.39702 PMC6428571

[evl3165-bib-0161] Speidel, L. , M. Forest , S. Shi , and S. R. Myers . 2019 A method for genome‐wide genealogy estimation for thousands of samples. Nat. Genet. 51:1321–1329. 31477933 10.1038/s41588-019-0484-x PMC7610517

[evl3165-bib-0162] Stahl, E. A. , G. Dwyer , R. Mauricio , M. Kreitman , and J. Bergelson . 1999 Dynamics of disease resistance polymorphism at the Rpm1 locus of Arabidopsis. Nature 400:667–671. 10458161 10.1038/23260

[evl3165-bib-0163] Steinrücken, M. , A. Bhaskar , and Y. S. Song . 2014 A novel spectral method for inferring general diploid selection from time series genetic data. Ann. Appl. Stat. 8:2203–2222. 25598858 10.1214/14-aoas764 PMC4295721

[evl3165-bib-0164] Sverrisdóttir, O. O. , A. Timpson , J. Toombs , C. Lecoeur , P. Froguel , J. M. Carretero , et al. 2014 Direct estimates of natural selection in Iberia indicate calcium absorption was not the only driver of lactase persistence in Europe. Mol. Biol. Evol. 31:975–983. 24448642 10.1093/molbev/msu049

[evl3165-bib-0165] Tajima, F. 1989 Statistical method for testing the neutral mutation hypothesis by DNA polymorphism. Genetics 123:585–595. 2513255 10.1093/genetics/123.3.585 PMC1203831

[evl3165-bib-0167] Torada, L. , L. Lorenzon , A. Beddis , U. Isildak , L. Pattini , S. Mathieson , et al. 2019 ImaGene: a convolutional neural network to quantify natural selection from genomic data. BMC Bioinformatics 20:337. 31757205 10.1186/s12859-019-2927-x PMC6873651

[evl3165-bib-0168] Turchin, M. C. , C. W. K. Chiang , C. D. Palmer , S. Sankararaman , D. Reich , Genetic Investigation of ANthropometric Traits (GIANT) Consortium , et al. 2012 Evidence of widespread selection on standing variation in Europe at height‐associated SNPs. Nat. Genet. 44:1015–1019. 22902787 10.1038/ng.2368 PMC3480734

[evl3165-bib-0169] Turner, S. 2014 qqman: Q‐Q and manhattan plots for GWAS data.

[evl3165-bib-0170] Turner, T. L. , and P. M. Miller . 2012 Investigating natural variation in Drosophila courtship song by the evolve and resequence approach. Genetics 191:633–642. 22466043 10.1534/genetics.112.139337 PMC3374323

[evl3165-bib-0171] van der Valk, T. , F. Lona Durazo , L. Dalén , and K. Guschanski . 2017 Whole mitochondrial genome capture from faecal samples and museum‐preserved specimens. Mol. Ecol. Resour. 17:e111–e121. 28675688 10.1111/1755-0998.12699

[evl3165-bib-0173] Wales, N. , and L. Kistler . 2019 Extraction of ancient DNA from plant remains Pp. 45–55 *in* Shapiro B., Barlow A., Heintzman P. D., Hofreiter M., Paijmans J. L. A., and Soares A. E. R., eds. Ancient DNA. Humana Press, New York. 10.1007/978-1-4939-9176-1_6 30875043

[evl3165-bib-0174] Wright, S. 1931 Evolution in Mendelian populations. Genetics 16:97–159. 17246615 10.1093/genetics/16.2.97 PMC1201091

[evl3165-bib-0175] Wright, S. 1948 On the roles of directed and random changes in gene frequency in the genetics of populations. Evolution. 2:279–294. 18104586 10.1111/j.1558-5646.1948.tb02746.x

[evl3165-bib-0176] Ye, K. , F. Gao , D. Wang , O. Bar‐Yosef , and A. Keinan . 2017 Dietary adaptation of FADS genes in Europe varied across time and geography. Nature Ecol. Evol. 1:1–11. 29094686 10.1038/s41559-017-0167 PMC5672832

[evl3165-bib-0177] Zeng, K. , Y. X. Fu , S. Shi , and C. I. Wu . 2006 Statistical tests for detecting positive selection by utilizing high‐frequency variants. Genetics 174:1431–1439. 16951063 10.1534/genetics.106.061432 PMC1667063

